# Positive and negative falls short: why any attempt to rename schizophrenia must include careful consideration of the sensori-/psychomotor domain

**DOI:** 10.1007/s00406-025-02108-7

**Published:** 2025-09-16

**Authors:** Dusan Hirjak, Sebastian Walther, Vijay A. Mittal

**Affiliations:** 1 Department of Psychiatry and Psychotherapy, Central Institute of Mental Health, Medical Faculty Mannheim, University of Heidelberg, 68159 Mannheim, Germany; 2 German Centre for Mental Health (DZPG), Partner site Mannheim, Mannheim, Germany; 3 Department of Psychiatry, Psychosomatics and Psychotherapy, Center of Mental Health, University Hospital of Würzburg, Würzburg, Germany; 4 Department of Psychology, Northwestern University, Evanston, IL 60208 United States; 5 Department of Psychiatry, Northwestern University, Chicago, IL 60611 United States

Dear Editor,

We read with great interest the editorial by Leucht et al. [1] proposing to rename schizophrenia as “Positive and Negative Symptoms Disorder (PND).” We commend the authors for their thoughtful and well-argued attempt to address the historical and conceptual problems associated with the term “schizophrenia,” as well as their commitment to stigma reduction and diagnostic clarity. While we truly appreciate the authors’ effort to rethink the name of schizophrenia, we believe the focus on positive and negative domains represents a missed opportunity, and that it may lead to the perpetuation of some longstanding issues that impact progress in the field. More specifically, while positive and negative symptoms are certainly important, they don’t capture the full complexity of the condition. Schizophrenia involves a wide range of experiences and behaviors, and reducing it to just these two symptom clusters risks turning a deeply multifaceted disorder into a checklist. If the name is going to reflect core symptom domains, then including sensori-/psychomotor and cognitive features is not just reasonable—it’s essential.

Below, we outline several points of disagreement with Leucht et al. [1] and explain why the sensori-/psychomotor domain should be recognized as an integral part of any revised conceptualization and naming of schizophrenia:

For over two centuries, sensori-/psychomotor and behavioral symptoms have been central to diagnosing schizophrenia and related psychotic disorders [2–5]. Early syndromes like melancholia, catatonia, hebephrenia, and dementia praecox were distinguished by characteristic motor behaviors and expressive changes. Hecker’s hebephrenia [6] involved disorganized, childlike behavior; Kahlbaum’s catatonia featured stupor and stereotypies; Kraepelin and Bleuler also emphasized motor signs. These clinical observations once shaped psychiatric diagnosis and remain relevant today, as modern technologies allow for increasingly precise and objective measurement of motor and behavioral abnormalities, linking them directly to underlying brain structure and function [7, 8].

Second, we were particularly surprised that, although the authors mentioned "motor" and "psychomotor" several times and acknowledged their relevance within diagnostic frameworks, they ultimately chose to exclude these domains—justifying this by associating them primarily with catatonia, a rationale that does not withstand current empirical or conceptual scrutiny. From a clinical standpoint, individuals with schizophrenia frequently display a broad spectrum of sensori-/psychomotor abnormalities, many of which extend well beyond the boundaries of catatonia as defined by ICD-11 [9, 10]. These include neurological soft signs (NSS), dyskinesia, dystonia, clumsiness, reduced physical activity, and impaired gestures, among others [11–13]. Sensori-/psychomotor phenomena in schizophrenia are not only well-characterized clinically but are also supported by neuroscientific research linking them to distinct neural correlates across motor, premotor, and cerebello-thalamo-cortical circuits [14–20]. Furthermore, these phenomena occur frequently, even in the absence of antipsychotic medications [21] or catatonic episodes, and they may precede [22, 23], co-occur with [24], or persist independently of positive and negative symptoms. Excluding them from the proposed new name risks reinforcing a fragmented and outdated understanding of the disorder.

Third, we were also concerned by the underlying assumption that only symptoms unique to schizophrenia deserve to be reflected in the name. As the authors argue against including sensori-/psychomotor and cognitive domains because they are “not unique” to schizophrenia, we would point out that delusions, hallucinations, and negative symptoms are also not exclusive to this disorder. By this logic, one would also have to remove hallmark features from consideration. Transdiagnostic features are not a disqualifier—they are often the most robust and clinically relevant dimensions in psychiatry. Indeed, systems such as RDoC and HiToP have gained increased attention, and these frameworks highlight the essential importance of considering unique and transdiagnostic elements [25]. It is noteworthy that RDoC has recently included a sensorimotor domain [26]. Along these lines, it is also important to highlight that motor features in particular can be useful for understanding distinct and transdiagnostic vulnerability, as some features are unique to psychosis and other carry across forms of serious mental illness [27–29]. The focus should not be on uniqueness, but on relevance, prevalence, and predictive utility.

Fourth, sensori-/psychomotor phenomena are known among the earliest vulnerability signs to appear in premorbid period, track and interact with human development in the premorbid period [30, 31]. Notably, a body of recent evidence suggest that they hold particular clinical relevance for the early identification of individuals at clinical-high risk (CHR) [32], for predicting illness trajectory [33], and for informing treatment strategies in schizophrenia [32]. Notably, brain regions implicated in psychomotor slowing may offer promising targets for non-invasive neuromodulation [34]. This sets the sensori-/psychomotor domain apart, as—beyond the domains of auditory-verbal hallucinations and cognitive impairment—there remains a striking lack of research exploring whether stimulation of specific neural circuits can effectively alleviate other core symptoms of the disorder.

Finally, we agree with Leucht et al. [1] that a name change is needed. However, simply doubling down on DSM/ICD-style symptom listings is unlikely to resolve the conceptual and practical issues surrounding the current term “schizophrenia.” If a new name is to fulfill its promise—clarity, acceptance, utility—it must reflect the multifaceted nature of the condition, including sensori-/psychomotor aspects. Although a detailed discussion lies beyond the scope of this letter, it is worth underscoring that cognitive and social domains are fundamental—not only diagnostically, but also for guiding treatment strategies and informing prognostic outlooks [35, 36]. Any serious effort to rename or reconceptualize the disorder should give these domains the careful consideration they deserve. We encourage the field to adopt a more integrative terminology that reflects both the clinical complexity of schizophrenia and the diverse lived experiences of those affected.

## Data Availability

No data was used for the research described in the article.
